# Examining the Conceptual and Measurement Overlap of Body Dissatisfaction and Internalized Weight Stigma in Predominantly Female Samples: A Meta-Analysis and Measurement Refinement Study

**DOI:** 10.3389/fgwh.2022.877554

**Published:** 2022-04-21

**Authors:** Jessica F. Saunders, Sarah Nutter, Shelly Russell-Mayhew

**Affiliations:** ^1^Hiatt School of Psychology, Clark University, Worcester, MA, United States; ^2^Department of Educational Psychology and Leadership Studies, University of Victoria, Victoria, BC, Canada; ^3^Werklund School of Education, University of Calgary, Calgary, AB, Canada

**Keywords:** body dissatisfaction, internalized weight stigma, internalized weight bias, measurement, concept proliferation

## Abstract

Both body dissatisfaction and internalized weight stigma have been identified as risk factors for many negative health outcomes for women, including depression and eating disorders. In addition to these contributions, these concepts have been found to overlap to various degrees in existing literature. We conducted a systematic review and meta-analysis on articles published prior to February 2022 to demonstrate the conceptual and measurement overlap between body dissatisfaction and internalized weight stigma as currently quantified. We identified 48 studies examining the interrelation between body dissatisfaction and internalized weight stigma in predominantly female samples. Stronger correlations between these two constructs, some bordering on multicollinearity, were prevalent in community samples compared to clinical samples and with some but not all the commonly used measures in the body image and weight stigma fields. Body mass index (BMI) moderated these relations such that individuals with higher self-reported BMI were more likely to report lower correlations between the constructs. This concept proliferation, stronger for individuals with lower BMIs and community samples, necessitates the need change how we conceptualize and measure body dissatisfaction and internalized weight stigma. To this end, we conducted study two to refine existing measures and lessen the degree of measurement overlap between internalized weight stigma and body dissatisfaction, particularly in community samples of women. We aimed to clarify the boundaries between these two concepts, ensuring measurement error is better accounted for. Female university students completed existing measures of body satisfaction and internalized weight stigma, which were analyzed using an exploratory followed by a confirmatory factor analysis. In our attempts to modify two existing measures of internalized weight stigma and body dissatisfaction, the majority of the internalized weight stigma items were retained. In contrast, most of the body dissatisfaction items either cross-loaded onto both factors or loaded on to the internalized weight stigma factor despite being intended for the body dissatisfaction factor, suggesting that the measurement issues identified in recent prior research may be due not only to the way we conceptualize and quantify weight stigma, but also the ways in which we quantify body dissatisfaction, across the existing corpus of body dissatisfaction scales.

## Introduction

Both body dissatisfaction and internalized weight stigma are robust predictors of disordered eating and the development of clinically significant eating disorders for women [EDs; (1, 2)]. As conceptualized and defined, body dissatisfaction and internalized weight stigma are two distinct concepts. Body dissatisfaction is defined as an individual's negative cognitions and emotions about their body (3). Consistently, body dissatisfaction emerges as the most potent risk factor for EDs for women (4). Body dissatisfaction also has wide-ranging effects on health outcomes, including decreased self-esteem and engagement in physical activity, and increased risk for depression (2, 5). In contrast, internalized weight stigma, also referred to as self-directed weight stigma, involves holding negative attitudes about oneself because of self-perceived excess body weight and devaluation of the self, based on societal pressures (6). Internalized weight stigma is also associated with numerous negative health outcomes, including depression (7), poorer psychological well-being (8), reduced engagement in physical activity (1), disordered eating behavior (9), and EDs (10, 11).

Given the similarities between these two concepts in definition as well as in associated health outcomes, it is important to examine if measurement tools are assessing each construct as intended, or if the lines between the measurement of body dissatisfaction and internalized weight stigma are blurred. Recently, Meadows and Higgs (12) examined the conceptual overlap of internalized weight stigma, body dissatisfaction, and self-esteem, the results of which suggested these constructs may be separate conceptualizations of a single factor. Adding to the confusion, Austen et al. (13) note the interchangeable use of terms to refer to internalized weight stigma, which can further blur boundaries between concepts if researchers are interchangeably using terms that were intended to hold distinct meaning.

## Conceptual Overlap: Concept Creep or Construct Proliferation?

Given these blurred boundaries and the array of terms used to describe internalized weight stigma, it is imperative to examine whether internalized weight stigma is a separate construct from body dissatisfaction, with error in existing measures contributing to conceptual overlap, or if they represent one construct with two distinct labels. In discussing the evolution of constructs, Haslam (14) noted that semantic shifts in our understanding of concepts over time can expand and blur the measurement boundaries between two or more concepts. According to Haslam (14), these shifts are referred to as concept creep, which occurs as concepts are continuously influenced by the changing social environment.

As hypothesized by Meadows and Higgs (12), however, the relationship between body dissatisfaction and internalized weight stigma may be better explained by reconceptualizing them as a single construct. In commenting on a perceived widespread issue with empirical redundancy in Industrial-Organizational (IO) psychology research, Le et al. (15) coined the term construct proliferation to describe the process by which distinct research streams build research around ostensibly unique, but empirically indistinguishable, constructs. Construct proliferation can impede the creation of knowledge by researchers from different research streams, preventing the collaboration and influence that may come from such partnerships (15, 16). Although construct proliferation was coined to describe empirical redundancies in IO research, scholars in other areas of psychology have acknowledged its utility in removing redundancies from psychological research overall (17). Regardless of whether the relationship between body dissatisfaction and internalized weight stigma suffers from concept creep or construct proliferation, a lack of clarity on the nature of their conceptual overlap will potentially have direct and cascading effects on research and clinical practice. Given the deleterious consequences of both internalized weight stigma and body dissatisfaction, it is critical both are measured accurately so clinicians and researchers target the necessary constructs in future interventions.

## Body Dissatisfaction and Internalized Weight Stigma: an Examination of Conceptual Overlap

In identifying ways to move an examination of conceptual overlap forward, Meadows and Higgs (12) suggested replicating their results with alternative measures of internalized weight stigma. Additionally, Austen et al. (13) noted a Delphi study with a panel of experts may provide an additional opportunity to add clarity to definitions and understandings of these constructs. We assert that, prior to replicating and extending the results of Meadows and Higgs (12), it may be useful to survey conceptual overlap within existing literature quantitatively. Further, while the Delphi method is a useful and rigorous method for consensus-building among experts and can contribute to clarifying issues in an area of research, it is a complex and time-consuming approach that involves surveying many experts as well as engaging in efforts to reduce bias in findings, due to the subjective nature of the method (18, 19). Further examination of the relationship between body dissatisfaction and internalized weight stigma quantitatively provides the opportunity to strengthen the foundation of evidence to support further investigation into potential concept creep or construct proliferation.

To this end, we conducted a meta-analysis of research examining internalized weight stigma and body dissatisfaction. After conducting the meta-analysis and identifying the measures of body dissatisfaction and internalized weight stigma that demonstrate the most and the least conceptual overlap, we conducted an empirical study in a sample of racially and ethnically diverse college-attending women. This study aimed to refine existing measures of body dissatisfaction and internalized weight stigma identified in the meta-analysis as suitable for community samples of women, while removing the conceptual overlap.

Although researchers have recently conducted a meta-analysis of internalized weight stigma and related body image correlates, including body dissatisfaction, positive body image, and weight control beliefs (20), we identified two important limitations of this meta-analysis that we seek to address in our current meta-analysis. First, in identifying papers that measured negative body image, Romano et al. (20) included papers that measured appearance, weight, or shape dissatisfaction as well as body shame, weight-contingent self-worth, body image flexibility, body image avoidance, self-objectification, and appearance anxiety. We assert that many, if not all, of these latter concepts may be regarded as distinct constructs (e.g., body shame and self-objectification: Fredrickson and Roberts, (21); McKinley and Hyde, (22)) and may not adequately quantify construct proliferation between body dissatisfaction and internalized weight stigma. Thus, to focus more narrowly on the concept of body dissatisfaction, we limited our analysis to papers measuring appearance, weight, or shape dissatisfaction. Second, given that researchers have examined internalized weight stigma in both clinical and community-based samples, but have yet fully to compare differences in the degree of internalized weight stigma in these populations, we were also interested in examining potential differences in clinical and community-based samples. Population-based differences were not examined in the analysis conducted by Romano et al. (20) but may further our understanding of which groups are most affected by the conceptual and measurement overlap.

## Study 1: Systematic Review and Meta-Analysis of Existing Literature

### Method

#### Search Strategy and Eligibility Criteria

**Figure 1:**
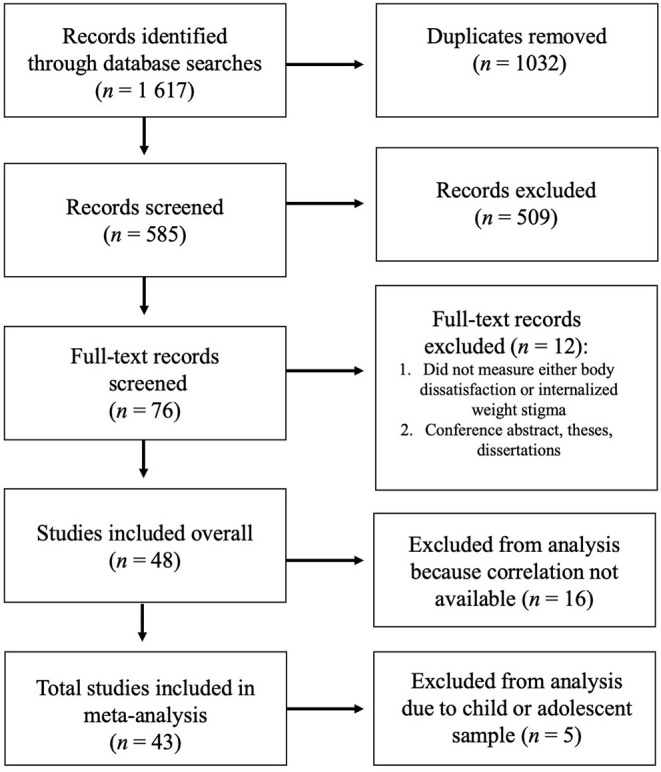
PRISMA diagram results from database searches through meta-analysis.

In conducting our search, we examined the search strategies of two previous systematic reviews on internalized weight stigma (1, 20), and utilized the search terms for internalized weight stigma that were first identified by Pearl and Puhl (1) and also used by Romano et al. [(20): weight bias internalization; weight bias internalisation; internalized weight bias; internalised weight bias; internalized weight stigma; internalised weight stigma; self-directed weight stigma; self-directed weight bias; weight self-stigma]. The search was conducted in seven databases on January 31, 2022, by the second author: PubMed, SCOPUS, Science Direct, EMBASE, PsychINFO, CINAHL, and Web of Science. Studies were included if they were written in English, tested human participants, quantitatively measured both body dissatisfaction (defined as measuring dissatisfaction with appearance, weight, and/or shape) and internalized weight stigma, and were peer reviewed. The search yielded 585 abstracts after the removal of duplicates (*n* = 1,032), which were screened by the first and second authors. Of the 76 papers that were identified for full-text screening, 48 were included in the systematic review and 43 in the meta-analysis. Given the small number of studies examining these constructs in child and adolescent samples (*n* = 5), we included these in the systematic review for descriptive purposes but not the meta-analysis. Papers were excluded from the systematic review and meta-analyses (*n* = 28) for the following reasons: (1) not measuring both constructs in question (*n* = 4), (2) conference abstracts, theses, and dissertations (*n* = 8), (3) or the correlation not being available from the study authors (*n* = 16). The search structure and process are summarized in Figure 1. Notably, we identified an additional 20 papers that were not included in the meta-analysis conducted by Romano et al. (20), two of which were published online after October 2021, when their search was conducted.

#### Meta-Analysis

A meta-analysis of the 43 identified articles and follow-up analyses of sub-groups (clinical vs. college/community) and of BMI as a potential moderator were conducted using *Meta-Essentials*, a set of excel workbooks for meta-analysis (23) to calculate the standard errors, moderators, and group-level differences and SPSS 28.0 to produce the forest plot. Data were independently extracted and analyzed by the first author. Given the potential heterogeneity in both samples and measurement, we conducted each meta-analysis using random effects models. Where studies included more than one measure of body dissatisfaction (two subscales of the MBSRQ or EDE-Q were administered), we averaged the correlations so as to not include each participant more than once. This choice was also made as some studies report this composite correlation within their manuscript. In instances where data from male and female or race sub-samples were analyzed separately, we entered the study in the meta-analysis twice (once for each sample). Q and *I*^2^ were calculated to determine heterogeneity, with *I*^2^ expressing the percentage of variability attributable to heterogeneity rather than sampling error. As such, possible *I*^2^ values range from 0 to 100% with higher values representing greater heterogeneity. We assessed publication bias by implementing Egger's test (24), which examines whether the intercept from the linear regression equation is significantly different from zero. A statistically significant intercept indicates that publication bias is likely present. We examined group differences between clinical and community samples as the manifestations of body dissatisfaction and internalized weight bias were likely to differ between these groups, and considered BMI as a moderator because again, the way these constructs interrelate may depend upon body size and the lived experiences associated with that body size.

### Results

#### Systematic Review

Within the 48 papers that examined the correlation between body dissatisfaction and internalized weight stigma, nine scales were used to assess body dissatisfaction in adults: the weight and shape concern subscales of the Eating Disorder Examination Questionnaire or Eating Disorder Examination [EDE-Q, (25, 26); *n* = 15], multiple different subscales of the Multidimensional Body-Self Relations Questionnaire [MBSRQ, (27); *n* = 13], the BSQ [(28); *n* = 6], the body dissatisfaction subscale of the Eating Disorder Inventory [EDI, (29); *n* = 2], the Body Uneasiness Test [(30); *n* = 1], the Male Body Attitudes Scale-Revised [(31); *n* = 1], the Weight Concerns Scale *n* = 1 (32), the body dissatisfaction subscale of the Eating Pathology Symptoms Inventory (EPSI (33); *n* = 1) and the Body Image States Scale [(34); *n* = 1]. Two adult studies and two youth studies used researcher-created item(s). The remaining youth studies used either the EDE-Q (*n* = 1) or the Physical Self-Description Questionnaire [PSDQ; (35); *n* = 1].

In adult and youth samples, internalized weight stigma was quantified by 10 different iterations of the WBIS: the original 11-item scale [(6); *n*= 18], the WBIS modified for all body weights with 11 items [WBIS-M; (1); *n* = 10], a 10-item version [(36); *n* = 4], a modified 10-item version (*n* = 1), the 19-item WBIS [(6); *n* = 2], a modified 9-item Italian version [(37); *n* = 1], a modified 10-item German version (34; *n* = 1), a modified Greek version (38), a modified Spanish version ((39); *n* = 1), and the WBIS for children (*n* = 2). The remaining adult studies quantified internalized weight stigma through administration of the Weight Self Stigma Questionnaire [(40); *n* = 6] and the self-stigma sub-scale of the Acceptance and Action for Weight Questionnaire [AAQ-W; (41); *n* = 1].

We divided the 48 studies into three groups: studies of individuals with clinically significant ED characteristics or those seeking treatment for an eating and or weight concern (*n* = 19, see Table 1), studies with community-based or college student samples (*n* = 29, see Table 2), and child/adolescent samples (*n* = 5, contained within Tables 1, 2), as the manifestations of body dissatisfaction and internalized weight stigma and the interrelation between these constructs were likely to differ between these groups.

**Table 1:** Systematic review of clinical samples.

**References**	**Measures**	**Sample**	**Correlation *(r)***
Almenara et al. (42)	EDE-Q; WSSQ	Women with BMI > 30 in outpatient treatment for weight issues	WSSQ and EDE-Q Weight =.32; WSSQ and EDE-Q Shape = 0.40
Burmeister et al. (43)	MSBRQ BASS subscale; 11-item WBIS	Adults in behavioral weight loss intervention	0.39
Burmeister and Carels (44)	14-item BSQ; 11-item WBIS	Adults seeking weight loss treatment	0.51
Carels et al. (45)	MBSRQ Appearance Evaluation subscale; 11-item WBIS	Overweight and obese recruited for weight loss intervention	0.48
Carels et al. (46)	MSBRQ BASS subscale; 11-item WBIS	Adults in behavioral weight loss intervention	0.37
Durso et al. (10)	EDE-Q; 11-item WBIS	BED patients	EDE-Shape and WBIS = 0.48 EDE-Weight and WBIS = 0.37
Durso et al. (47)	14-item BSQ; 11-item WBIS	Overweight and obese treatment seeking adults	0.66
Eisenberg et al. (48)	Researcher created body size and shape satisfaction items 11-item WBIS;	Overweight adults seeking weight counseling	White women Body Size Dissatisfaction = 0.56 White women Body Shape Satisfaction = 0.38 Black women Body Size Dissatisfaction = 0.29 Black women Body Shape Dissatisfaction = 0.38
Hübner et al. (49)	MBSRQ Appearance Evaluation and Appearance Orientation; 10-item WBIS	Pre-bariatric surgery patients	Appearance Evaluation and WBIS = 0.36 Appearance Orientation and WBIS = 0.29
Innamorati et al. (37)	BUT; 9-item WBIS	Italian overweight and obese patients in treatment for weight concerns	0.77
Lawson et al. (50)	EDE-Dissatisfaction; 11-item WBIS	Sleeve gastrectomy patients seeking treatment for eating/weight concerns	0.45
Lawson et al. (51)	EDE-Q Brief Overvaluation of Weight and Shape; 11-item WBIS	Adults seeking bariatric surgery	0.56
Lin and Lee (52)	MBSRQ Appearance Evaluation, and BASS subscales; Chinese WSSQ	Chinese overweight and obese adults	AE and WSSQ = 0.40 BASS and WSSQ = 0.41
Roberto et al. (53)^*^	EDE-Q Shape and Weight Concern; 10-item WBIS	Obese adolescents seeking bariatric surgery	EDE-Q Shape Concern = 0.82; EDE-Q Weight Concern = 0.55
Schvey et al. (54)	EDE-Q Shape and Weight Concern; 19-item WBIS	Adults meeting binge and purge behavioral criteria for an ED	EDE-Q Shape Concern = 0.72; EDE-Q Weight Concern = 0.66
Sevincer et al. (55)	EDE-Q; WSSQ	Severely obese outpatients (BMI > 35)	EDE-Shape and Self Devaluation = 0.23 EDE-Weight and Self Devaluation = 0.23 EDE-Shape and Enacted Stigma = 0.30 EDE-Weight and Enacted Stigma = 0.31
Wagner et al. (56)	8-item BSQ; 10-item WBIS	Patients presenting for bariatric surgery	0.70
Wang et al. (57)	EDE; 11-item WBIS	Patients with BED and obesity responding to treatment ad	0.59
Weineland et al. (58)	EDE-Q Weight and Shape Concern; AAQ-W Self-Stigma	Bariatric surgery patients	EDE-Q Weight Concern = 0.55; EDE-Q Shape Concern = 0.62

The asterisk (*) symbol indicates a child/adolescent sample.

**Table 2:** Systematic review of community samples.

**References**	**Measures**	**Sample**	**Correlation (*r*)**
Aim et al. (59)	BASS subscale of MBSRQ; 11-item WBIS-M	Emerging adults	0.63
Argyrides et al. (38)	MSBRQ Appearance Evaluation; Greek WBIS-M	Community sample in Greece	0.70
Austen et al. (60)	Male Body Attitudes Scale; 11-item WBIS-M	Internet panel	0.73
Bevan et al. (61)	MSBRQ Appearance Evaluation; 11-item WBIS-M	University students	0.71
Boswell and White (62)	EDE-Q Weight and Shape Concern; 11-item WBIS	Overweight and obese adults	Women: EDE-Shape = 0.71 Women: EDE-Weight = 0.66 Men: EDE-Shape and WBIS = 0.74 Men: EDE-Weight and WBIS = 0.68
Burnette and Mazzeo (63)	EDE-Q Dissatisfaction; 11-item WBIS-M	College women in intuitive eating trial	0.85
Burnette and Mazzeo (64)	EDE-Q Dissatisfaction; 11-item WBIS-M	College students	0.80
Carels et al. (65)	Weight Concerns Scale; 11-item WBIS-M	Women in heterosexual relationship	0.64
Durso and Latner (6)	14-item BSQ; 11-item WBIS	Overweight internet sample	0.75
Gmeiner and Warschburger (66)^*^	Researcher-created single-item of body dissatisfaction; WBIS-C	Children between ages of 6-11	0.36
Godoy-Izquierdo et al. (67)	Researcher-created single-item of body dissatisfaction; WBIS-M (Spanish)	Spanish adults w/BMI > 25	0.44
Horn and Jongenelis (68)	EDI Body Dissatisfaction; 11-item WBIS-M	Prolific panel members	0.72
Jung et al. (69)	Appearance evaluation subscale of MSBRQ; 11-item WBIS	Randomly selected Germans	0.40
Lee et al. (70)	Appearance Evaluation subscale and BASS of MBSRQ combined; 11-item WBIS	College student sample	0.67
Lin et al. (71)^*^	Real/ideal discrepancy; Self-devaluation sub-scale of the WSSQ	Overweight and obese adolescents	0.32
Maiano et al. (72)^^*^^	Physical Appearance Self-Description Questionnaire and self-stigma subscale of the WSSQ	Overweight and obese French-speaking adolescents	0.43
Meadows and Higgs (12)	Appearance Evaluation subscale of MSBRQ and 11-item WBIS	Overweight and fat identifying participants	0.80
Meadows et al. (73)	Appearance Evaluation subscale of MSBRQ and self-stigma subscale of WSSQ	University student sample and MTurk sample	University sample = 0.68; MTurk sample = 0.55
Mensinger et al. (74)	EDE-Q Shape and Weight Concern and 11-item WBIS	Women in healthy living program	EDE-Q Shape = 0.36; EDE-Q Weight = 0.46
Olson et al. (75)	14-item BSQ; 10-item WBIS	Overweight and obese women seeking weight loss	0.54
Pearl et al. (36)	MBSRQ Appearance Evaluation; 10 item WBIS	WW participants in the U.S.	. 0.70
Pearl and Puhl (76)	14-item BSQ; 11-item WBIS	M-Turk across all weight statuses	0.77
Pearl and Dovidio (77)	EDI-BD; 11-item WBIS	Overweight M-Turk participants	0.69
Purton et al. (78)	EDE-Q (Weight and Shape Averaged); 11-item WBIS-M	Ethnically diverse college students	0.76 female 0.60 male
Romano et al. (79)	EPSI Body Dissatisfaction; 11-item WBIS-M	Two samples of college students	0.74, 0.76
Schvey and White (11)	EDE-Q; 19-item WBIS	Normal and underweight community members	EDE-Shape and WBIS = 0.67 EDE-Weight and WBIS = 0.69
Selensky and Carels (80)	Body Image States Scale (BISS); 11-item WBIS-M	Female college student volunteers for online experiment	0.27
Sienko et al. (9)	EDE-Q; 11-item WBIS	College students	EDE-Shape and WBIS = 0.77 EDE-Weight and WBIS = 0.80
Zuba and Warschburger (81)^*^	Single-item body dissatisfaction; WBIS-C	Children and adolescents 9-13 years old	.59

*EDE-Q, Eating Disorders Examination Questionnaire; WBIS, Weight Bias Internalization Scale; BSQ, Body Shape Questionnaire; EDI-BD, Eating Disorder Inventory-Body Dissatisfaction; EPSI, Eating Pathology Symptom Inventory; MSBRQ, Multidimensional Body Self Relations Questionnaire*.

* *child or adolescent sample*.

##### Clinical Samples

Participants in these 19 studies were either bariatric surgery patients (*n* = 6), in an outpatient weight loss clinical intervention (*n* = 10), or patients clinically identified as having binge eating disorder or bulimia (*n* = 3). Across all measures of body dissatisfaction and internalized weight stigma, the reported correlations ranged from 0.23 to 0.82 (see Table 1). The MBSRQ (*n* = 5) and EDE-Q/EDE (*n* = 9) were the most administered measures of body dissatisfaction among clinical samples. Five studies found moderate to strong correlations between WBIS or WSSQ scores and scores on the: (a) appearance evaluation sub-scale [0.36-0.40; (49, 52)]; (b) appearance orientation subscale [0.29-0.48; (45, 49, 52)]; and (c) the body areas satisfaction subscale [0.37-0.41; (43, 46, 52)] of the MBSRQ. The EDE and EDE-Q weight and shape concern subscales also correlated moderately to strongly, but not redundantly, with the WSSQ [0.23-0.40; (42, 55)] and stronger still with the 11-item WBIS [0.37-0.72; (10, 50, 51, 54, 57)]. Scores on the BSQ were strongly related to scores on the WBIS [0.51-0.66; (44, 47)] and the self-stigma subscale of the AAQ-W [0.70; (58)]. Finally, scores on the Body Uneasiness Test were very strongly correlated with scores on a revised, 9-item Italian version of the WBIS [0.77; (37)].

##### Community and College Samples

Participants in these 25 studies were either recruited from internet survey platforms (*n* = 13) or were college students (*n* = 9). Participants in these samples ranged in reported body mass index and ethnicities and were predominantly female. Within these samples, body dissatisfaction was either measured using the BSQ, EDE-Q, MSBRQ, EDI-BD, and EPSI scales or subscales or by a researcher created single-item. Internalized weight stigma was measured with the WBIS (n =12) or WBIS-M (n = 12), with one study using the self-stigma subscale of WSSQ (73). These numbers somehow were not updated in the most recent version but this reflects what is indicated in Table 2. Across all measures of body dissatisfaction and WBIS scores, the reported correlations ranged from 0.27 to 0.85 (see Table 2). Scores on the weight and shape concern subscales of the EDE-Q were very strongly correlated with scores on the WBIS-M [0.60-0.85; (63, 64, 78)] and WBIS [0.36-0.80; (9, 11, 62, 74)]. The reported correlations between BSQ scores and WBIS scores [0.54-0.77; (6, 75, 76)], and EDI-BD and WBIS scores [0.69-0.72; (68, 77)] were of similar strengths. The other measures used in community and college samples were MSBRQ subscales correlated with various iterations of the WBIS [0.40-0.80; (12, 36, 38, 59, 61, 69, 70)], the Male Body Attitudes Scale and 11-item WBIS-M [0.73; (60)], the body dissatisfaction subscale of the EPSI and the WBIS-M [0.74-0.76; (79)] and the Body Image States Scale correlated with the 11-item WBIS-M [0.27; (80)]. All but four of the published community or college-based studies we identified reported correlations exceeding a large effect size, some approaching a perfect linear relationship (82).

##### Child and Adolescent Samples

There was one clinical adolescent sample (53), where the EDE-Q and 10-item WBIS were administered yielding correlations ranging from 0.55 to 0.82. Gmeiner and Warschburger administered a researcher-created single-item to quantify body dissatisfaction and the WBIS-C and found a moderate correlation (*r* = 0.36) in a sample of children between the ages of six and 11. The correlation increased to 0.59 in a sample of children and adolescents between the ages of 9-13 (81). Lin et al. (71) and Maïano et al. (72) also found moderate correlations (*r*'s = 0.32 and 0.43, respectively) in samples of overweight and obese adolescents.

#### Meta-Analyses

The meta-analysis included 48 individual studies [the samples in 55 and 54 were split by gender; the samples in Eisenberg et al. (48) were split by race; and 66 and 51 both featured two studies]. Given the small number of studies with a youth sample, these studies were excluded from the meta-analysis. Combined, these 48 studies featured 42,128 participants. For the entire sample, the random effects model with a 95% confidence interval yielded a correlation of *r* = 0.61, 95% CI[0.57, 0.65], *Z* = 20.97, *p* < 0.001. The Q and *I*^2^ statistics indicated considerable heterogeneity across studies (Q = 869.41, *I*^2^ = 94.59%). We hypothesized that this heterogeneity may be due to the two types of sub-samples under investigation; thus, we proceeded with the sub-group analysis.

**Figure 2:**
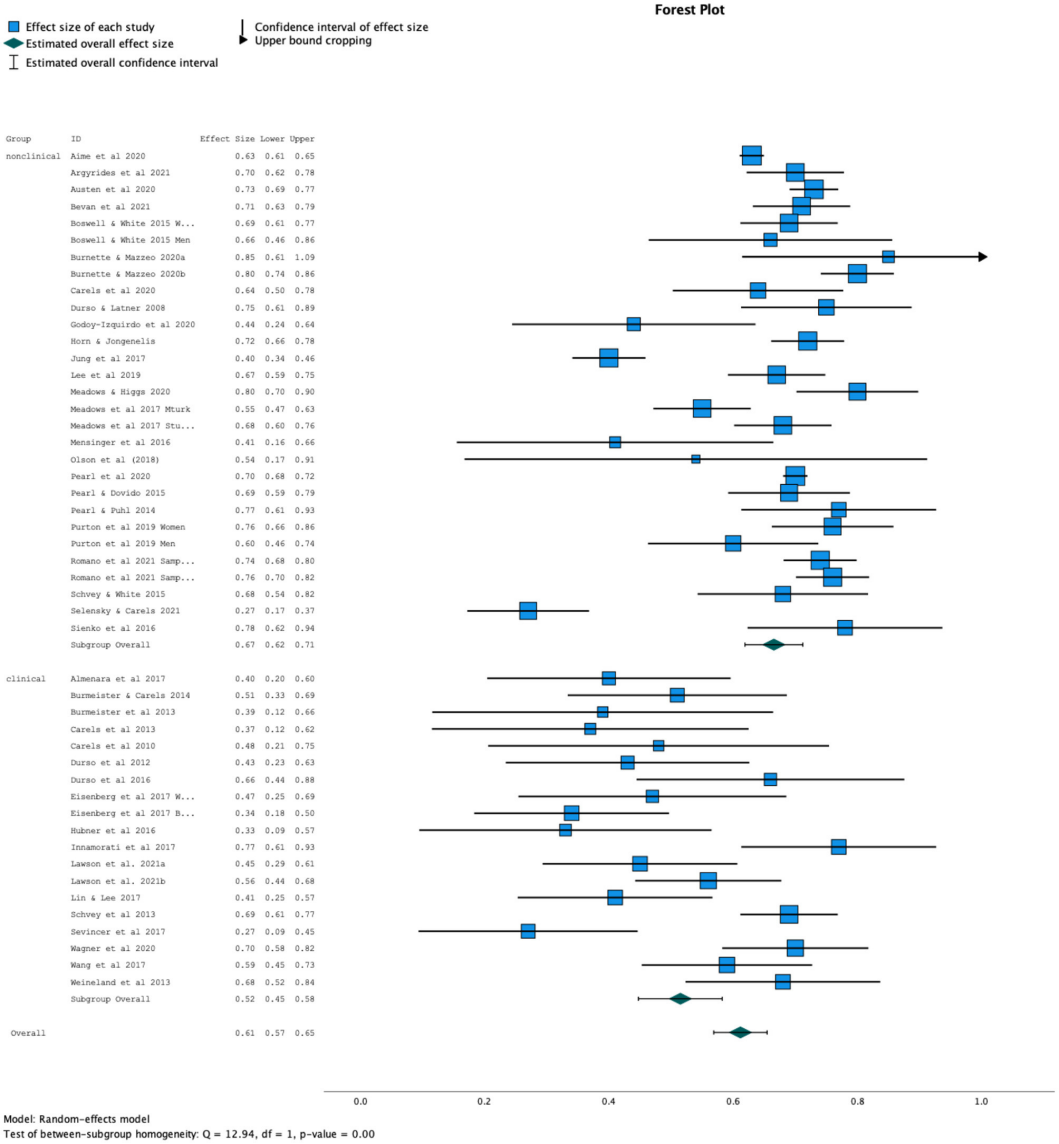
Forest plot by sub-group (clinical vs. community) demonstrating stronger correlation in community vs. clinical group.

Both the variance within groups [Q_within_ (54) = 69.30, *p* = 0.02] and variance between groups [Q_between_ (1) = 17.89, *p* < 0.001) were significant, suggesting significant differences in the strength of the correlation between clinical and community samples, and also significant variability within each group. As illustrated in Figure 2, the estimated effect size for the clinical samples was calculated at *r* = 0.52, 95% CI [0.45, 0.58]. In contrast, the estimated effect size for the community and college samples was calculated at *r* = 0.67, 95% CI [0.62, 0.71]. There was more heterogeneity in the community and college samples (Q = 634.40, *I*^2^ = 95.6%) compared to the clinical samples (Q = 132.34, *I*^2^ = 86.6%), which were more homogeneous.

**Figure 3:**
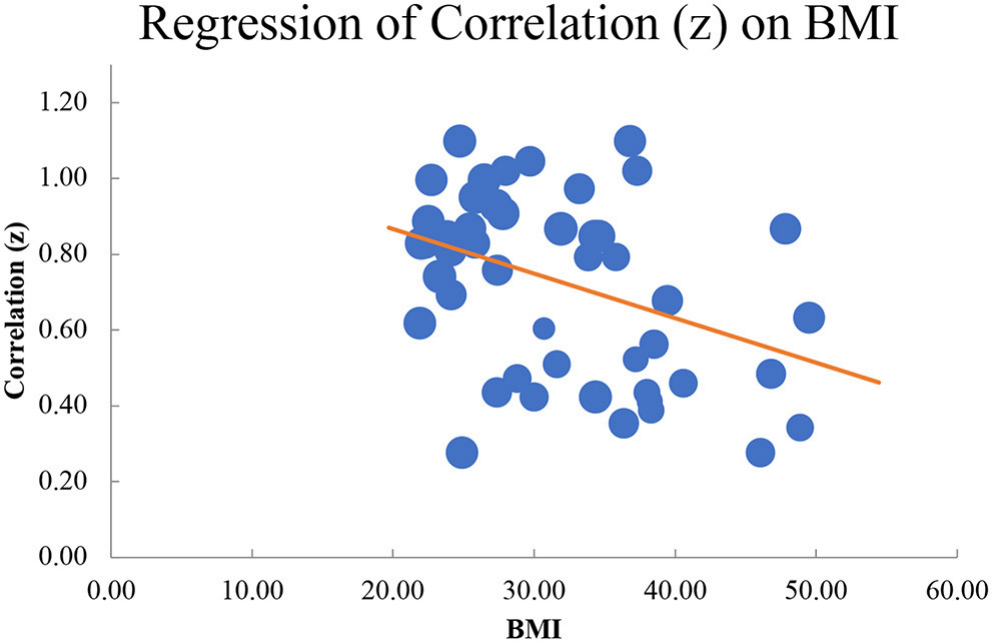
Moderation by BMI, indicating a stronger relation between body dissatisfaction and internalized weight stigma for those of lower weight.

**Figure 4:**
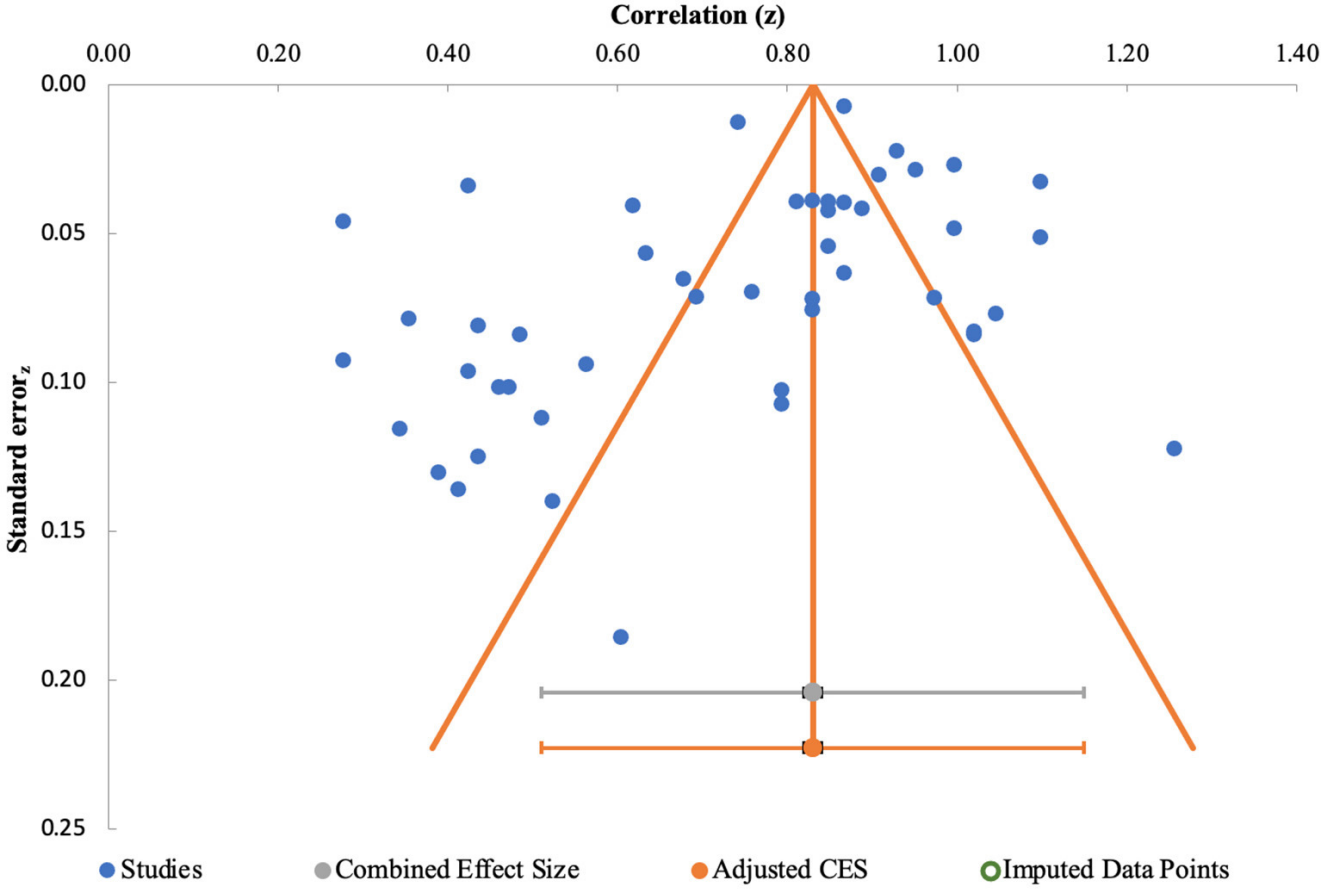
Funnel plot assessing publication bias using Egger's test.

Finally, we conducted a moderation analysis to examine the role of BMI in the relation between body dissatisfaction and internalized weight stigma. We extracted the mean BMI of each sample and entered these averages into the meta-regression. The random effects model yielded a significant moderation effect, β = −0.39, *Z* = −3.41, *p* < 0.001 (see Figure 3). This significant moderation effect indicates that the relation between body dissatisfaction and internalized weight stigma is highest for individuals with lower BMI and decreases in strength as BMI increases. Egger's test of publication bias indicated that no publication bias was identified, y-intercept = −1.73, 90% CI [−3.61, 0.15], *t* = −1.88, *p* = 0.07 (see Figure 4).

### Discussion

Body dissatisfaction and internalized weight bias are conceptualized as distinct concepts. Body dissatisfaction is defined as an individual's negative cognitions and emotions about their body (3), whereas internalized weight stigma, also referred to as self-directed weight stigma, involves holding negative attitudes about oneself because of self-perceived excess body weight and devaluation of the self, based on societal pressures (6). Despite these definitions, the meta-analyses yielded a significant, large correlation between body dissatisfaction and internalized weight stigma across all 48 studies, with significantly smaller correlations identified in clinical compared to community and college samples, and only moderate correlations identified in the five child and adolescent studies. The correlation between body dissatisfaction and internalized weight stigma in community and college samples bordered on multicollinearity in several instances, and was strongly but not redundant in clinical samples. Significant variability was identified within the clinical and community groups. Body mass index moderated the relation between body dissatisfaction and internalized weight stigma; the relation between body dissatisfaction and internalized weight stigma was the strongest for individuals with a lower BMI and weakened as average sample BMI increased.

These results extend on the recent findings by Romano et al. (20) in three important ways. First, while our meta-analysis resulted in a similar overall correlation between internalized weight stigma and body dissatisfaction as found by Romano et al. (20), our results suggest that there are significant differences between clinical and community/college populations, which was not examined in the existing literature. We also examined the relation between body dissatisfaction and internalized weight stigma developmentally, with our systematic review yielding consistently lower correlations in the youth samples compared to the adult samples. This finding suggests that the potential measurement issues are limited to adulthood and the measures used in childhood and adolescence are more distinct. Finally, our moderation analyses suggested that BMI was a significant moderator of the association between these constructs, which was examined but not discussed in the paper by Romano et al. (20). Given our calculated overall correlation between body dissatisfaction and internalized weight stigma approximated that found by Romano et al., it is possible that highly redundant correlations would again be found in community samples if we examined the related constructs of body shame, weight-contingent self-worth, body image flexibility, body image avoidance, self-objectification, and appearance anxiety that were included in the Romano paper. Future research should examine whether construct overlap between these distinct, yet related constructs, and internalized weight stigma is present among community samples to further improve measurement in the body image field.

There are several potential explanations for the differences observed between clinical and community samples in the extant literature. First, it may be that, as eating pathology severity becomes clinically significant, the strength of the relation between the concepts of internalized weight stigma and body dissatisfaction weakens, because other relevant clinical indicators are related to one or both concepts. A second possible explanation is that this difference relates to individual body weight, or body mass index (BMI). The studies to date with clinical samples have all examined these constructs in individuals in larger bodies seeking medically-supervised weight loss or psychiatric treatment for binge eating disorder, whereas the community samples typically include individuals with a wide range of BMIs. We explored this possibility without an a priori hypothesis, because prior research has shown that both body dissatisfaction and internalized weight stigma occur regardless of actual or perceived body weight (11), and that BMI does not consistently correlate with internalized weight stigma (49). Moreover, the correlation between internalized weight stigma and body dissatisfaction consistently remains strong even when controlling for BMI in the statistical model (76). However, we found a strong, negative effect of BMI across the 48 existing studies, suggesting that as BMI increases, the strength of the relation between body dissatisfaction and internalized weight bias decreases. This finding is contrary to what one may have expected, as it would follow theoretically that internalized weight stigma might have a greater negative impact on the body dissatisfaction of individuals who live in larger bodies and are exposed to greater rates of weight stigma and discrimination in their community.

A third explanation for the differences identified between clinical and community samples surrounds the measurement of these two constructs, particularly for individuals with lower BMIs. Although all of the measures administered in each study demonstrated acceptable psychometric properties, our examination of the interrelations between body dissatisfaction and internalized weight stigma identified (a) measures that approach multicollinearity with one another in both clinical and college or community samples (EDE-Q and WBIS), (b) measures that are very highly correlated with one another in community samples and highly, but not redundantly, correlated in clinical samples (BSQ and WBIS), and (c) measures that have, to date, only been administered in clinical samples but may show promise in quantifying body dissatisfaction and internalized weight stigma in community samples (subscales of the MBSRQ correlated with WBIS; WSSQ).

Only the WBIS or WBIS-M were used to quantify internalized weight stigma in the college and community samples, aside from one study by Meadows et al. (73) that used the self-stigma subscale of WSSQ in a sample of people self-identifying as living in larger bodies. This is likely a function of the fact that the scale has been normed with individuals across the weight spectrum (76), and was the most psychometrically-sound option of the existing measures for community samples with individuals of varying body sizes. Example items of the WBIS include “*I am less attractive than most other people because of my weight*” and “*my weight is a major way that I judge my value as a person*.” The correlations between body dissatisfaction and WBIS scores were quite high in each of the community and college samples, regardless of the measure of body dissatisfaction administered. In their meta-analysis, Romano et al. (20) indicate that the inclusive language of the WBIS-M scale may contribute to its redundant correlations with body dissatisfaction, arguing that “the weight-neutral language of [the WBIS-M], which is important from a weight stigma reduction and inclusivity perspective, may also draw out aspects of individuals' negatively valenced body image to a greater extent than is evident when the term “overweight” is used” (p. 8). The one exception to the high correlations with the WBIS and WBIS-M was seen in the data from Selensky and Carels (80) who used state, rather than trait-based measures.

On the other hand, the WSSQ was more regularly administered to clinical samples and consistently demonstrated moderate correlations with body dissatisfaction measures. The WSSQ may be a stronger indicator of internalized weight stigma not only in clinical samples, but also in community and college samples. This suggestion aligns with the findings of Meadows et al. (73) who found strong, but not redundant correlations between the WSSQ and the Appearance Evaluation subscale of the MSBRQ in a community sample of MTurk participants. We recommend that future research examine the psychometric properties of the WSSQ across weight statuses to ascertain if it is a psychometrically sound measure for quantifying internalized weight stigma in lower weight individuals. This would allow for the examination of the interrelations between WSSQ and body dissatisfaction scores in community or college samples to confirm whether these moderate correlations replicate in non-clinical groups. Likewise, several of the various MBSRQ sub-scales were only administered to the clinical samples, and these may be a strong alternative when assessing body dissatisfaction and in college and community samples.

The redundant correlations between body dissatisfaction and internalized weight stigma, particularly for the community samples may also have occurred because some items included within both body dissatisfaction and internalized weight stigma measures more strongly relate to conceptualizations of the other concept, thus blurring concept boundaries and contributing to construct proliferation for all samples. One recent study (78) found that, while highly correlated with one another, body dissatisfaction scores were not significant predictors of quality-of-life scores in college students, whereas internalized weight stigma scores were significant predictors. This finding suggests that there are nuances in the predictive power of scores on these measures and the amount of variance these constructs explain in certain outcomes. However, the extremely strong correlations between body dissatisfaction and internalized weight stigma in the community and college samples may point to a larger measurement problem: perhaps the commonly used measures of body dissatisfaction and internalized weight stigma among community samples of women are not measuring their intended constructs. This was a question we sought to explore in Study 2.

## Study 2: Examining and Removing Conceptual Overlap From Measures of Body Dissatisfaction and Internalized Weight Stigma

Given our findings in study one, we conducted study two to refine existing measures and lessen the degree of measurement overlap between internalized weight stigma and body dissatisfaction, particularly in community samples of women. In refining these measures, we aimed to clarify the boundaries between these two concepts, ensuring measurement error is better accounted for (16), and produce new measures of each construct for use in research and clinical practice. Such an improvement to existing measures would allow researchers and clinicians to better tease apart the interactive and additive roles of body dissatisfaction and internalized weight stigma on disordered eating and other psychological outcomes for women, and to design strong prevention programs specifically tailored to each of these risk factors.

### Method

The data are a part of a larger unpublished measurement validation study examining weight stigma and body image. After learning about the study requirements and providing informed consent, female participants (*n* = 492) provided demographic information and completed the standardized measures. All undergraduate Psychology majors over the age of 18 were eligible to participate; given the differing ways body dissatisfaction manifests by gender, we limited our current sample to female-identifying participants. Participation occurred online *via* the Qualtrics survey platform in exchange for course credit. All procedures were approved by the Institutional Review Board at a large, urban institution in the southeastern US.

#### Participants

The sample was predominantly Hispanic or Latina (66.5%; *n* = 327), followed by 14% Black (*n* = 69), 11.4% White (*n* = 56), 2.6% Asian (*n* = 13), 2.6% Biracial or bi-ethnic (*n* = 13), 0.8% Middle Eastern (*n* = 4), 0.4% South Asian (*n* = 2), 0.4% Alaskan or Pacific Islander (*n* = 2), 0.2% Southeast Asian (*n* = 1), and 1% Other (*n* = 5). Participants, all enrolled in an undergraduate Psychology degree, ranged in age from 18 to 54 (*M* = 22.98, *SD* = 5.57).

#### Measures

##### WBIS-M

As the modified WBIS is the only existing measure of internalized weight stigma that has been validated in college samples as well as in samples across the weight spectrum, we administered the WBIS-M (76) with the goal of refining the scale to better quantify weight stigma. The WBIS-M consists of 11 items rated on a six-point scale ranging from strongly disagree to strongly agree, with higher scores indicative of higher levels of internalized weight stigma. The Cronbach's α for the WBIS-M was 0.83.

##### BASS

The results of our meta-analysis indicated that the smallest amount of conceptual overlap between internalized weight stigma and trait body dissatisfaction consistently occurred when body dissatisfaction was quantified by the BASS subscale of the MSBRQ (27). Thus, we administered the BASS for measure refinement in the current study. The BASS consists of nine items rated on a five-point scale, ranging from very dissatisfied to very satisfied. We reversed the coding of each item, so a higher score was indicative of greater body dissatisfaction. The scale quantifies dissatisfaction with one's face (facial features and complexion), hair (color, thickness, and texture), lower torso (buttocks, hips, thighs, and legs), mid torso (waist and stomach), upper torso (chest, breasts, shoulders, arms), muscle tone, weight, height, and overall appearance. The Cronbach's α for the BASS was 0.83.

##### Data Analysis

To adequately refine the existing measures of internalized weight stigma and body dissatisfaction, we first split the sample into two random halves to allow for both an exploratory factor analysis (EFA) and confirmatory factor analysis (CFA) with the sample. We then conducted an EFA with the 11 items from the WBIS-M and the nine items from the BASS. As we were seeking two distinct scales, one that quantifies internalized weight bias and one that quantifies body dissatisfaction, we fixed the number of factors extracted to two. As internalized weight stigma and body dissatisfaction are likely to correlate even once the conceptual overlap has been removed, a promax oblique rotation was applied. Upon establishing which items to retain and which to remove based on EFA factor loadings, we conducted a CFA with the retained items and the other half of the sample.

### Results

There was a very small amount of missing data (0.3-0.7% of the BASS items) that were missing completely at random, Little's $\chi_{\left(2,227\right)}^{2}$ = 2311.68, *p* = 0.103. Missing data were imputed using expectation maximization in SPSS 26.0. The EFA yielded a Kaiser-Meyer-Olkin Measure of Sampling Adequacy value of 0.94 (seeking values >0.80) and a significant Bartlett's test of sphericity, indicating the data were suitable for an EFA. The two factors extracted explained 56.33% of the variance in the 20 items. As indicated in Table 3, the first item in the WBIS-M (*Because of my weight, I am just as competent as anyone*) cross-loaded on both factors, and was not retained as an item in our revised WBIS-M. This aligns with prior research using the WBIS (49). On the BASS, dissatisfaction with the lower torso, muscle tone, and overall appearance each cross-loaded on the two factors. The dissatisfaction with weight and dissatisfaction with mid-torso items loaded on to the internalized weight stigma factor and were eliminated. We retained four items on the BASS and 10 items on the WBIS-M. The retained items from the BASS quantified dissatisfaction with height, face, upper torso, and hair. All the other items demonstrated statistical overlap with the retained WBIS-M items. The scales were significantly but not redundantly correlated with one another, *r* = 0.28, 95% CI [0.16, 0.39], *p* < 0.001 when the redundant items were removed. Before removal of the five BASS and one WBIS-M item, the correlation between the scales was *r* = 0.69, 95% CI [0.62, 0.75], *p* < 0.001.

**Table 3:** Exploratory factor analysis factor loadings of BASS and WBIS-M items.

**Item**	**Internalized weight stigma loading**	**Body dissatisfaction loading**
^*^Face (facial features, complexion)	0.29	**0.69**
^*^Hair (color, thickness, texture)	0.13	**0.53**
^*^Lower torso (buttocks, hips, thighs, legs)	0.55	**0.64**
^*^Mid torso (waist, stomach)	0.73	0.53
^*^Upper torso (chest, breasts, shoulders, arms)	0.46	**0.72**
^*^Muscle tone	0.59	0.58
^*^Weight	0.81	0.55
^*^Height	0.11	**0.50**
^*^Overall appearance	0.72	0.73
Because of my weight, I feel that I am just as competent as anyone	0.31	0.12
**I am less attractive than most other people because of my weight**	**0.84**	0.49
**I feel anxious about my weight because of what people might think of me**	**0.80**	0.31
**I wish I could drastically change my weight**	**0.82**	0.40
**Whenever I think a lot about my weight, I get depressed**	**0.87**	0.44
**I hate myself for my weight**	**0.86**	0.44
**My weight is a major way that I judge my value as a person**	**0.77**	0.33
**I do not feel that I deserve to have a really fulfilling social life as long as I have a higher weight**	**0.70**	0.37
**I am OK having the weight that I have**	**0.79**	0.29
**Because of my weight, I do not feel like my true self**	**0.84**	0.40
**Because of my weight, I do not understand how anyone attractive would want to date me**	**0.84**	0.39

* Bold items were retained and items with an asterisk (*) are body dissatisfaction (BASS) items.

We next conducted a CFA with the other half of the sample (*n* = 246) to test the model fitness of the two factors. We allowed for error terms to correlate within the internalized weight stigma scale. The data demonstrated univariate normality with no skewness or kurtosis values greater than the absolute value of 1.3. The data did demonstrate multivariate non-normality (kurtosis = 41.21) and subsequent analyses were bootstrapped with 1,000 resamples to account for the non-normality. The 14-item model (10 items on the WBIS factor and 4 on the BASS factor) fit the data well: CFI = 0.97, TLI = 0.96, RMSEA = 0.05, 90% CI [0.04, 0.07], p-close = 0.17. All items loaded significantly onto their respective factor. Standardized factor loadings ranged from 0.38 to 0.66 for the BASS items and 0.56 to 0.88 for the WBIS items.

### Discussion

In our attempts to modify two existing measures of internalized weight stigma and body dissatisfaction, the majority of the internalized weight stigma items (10 out of 11) loaded strongly onto the internalized weight stigma factor, with one item cross-loading on both the internalized weight stigma and body dissatisfaction factors. This same item showed low inter-item correlations in a prior scale translation and validation study (83). In contrast, the majority of the body dissatisfaction items (five out of nine) either cross-loaded onto both factors or loaded on to the internalized weight stigma factor despite being intended for the body dissatisfaction factor. The two scales resulting from the EFA and CFA consisted of 10 items quantifying internalized weight stigma and four items quantifying body dissatisfaction. None of the retained body dissatisfaction items referred to weight or size of body parts. This suggests that the measurement issues identified in recent prior research (12, 79) may be due not only to the way we conceptualize and quantify internalized weight stigma, but also the ways in which we conceptualize and quantify body dissatisfaction, across the existing corpus of body dissatisfaction scales.

For example, many of the BSQ items are directly related to being “fat,” rather than referring to body dissatisfaction more generally (e.g., “*has being undressed, such as when taking a bath, made you feel fat?*”). Similarly, half of the items in the body dissatisfaction subscale of the EDI refer to the self-perception that one is “too large.” Given these size-focused item, it is important to consider whether these items truly quantify body dissatisfaction. Rather than quantifying an individual's negative cognitions and emotions about their body (3), these items may be described as more accurately measuring an individual's negative attitudes about oneself because of self-perceived excess body weight, or internalized weight stigma (6). Although the existing body dissatisfaction measures demonstrate strong psychometric properties (84), these measures may be unintentionally measuring internalized weight stigma simultaneously along with body dissatisfaction. Although the BASS and WBIS-M showed the smallest correlations in our meta-analysis (study 1), many of the items from the BASS appear to be measuring internalized weight stigma in study 2. Given this conceptual overlap, we suggest using a modified BASS in clinical and research situations where it is important to measure the distinct effects of body (dis)satisfaction and internalized weight stigma.

## General Discussion and Suggestions for Future Research

Overall, the results of this research are consistent with previous findings that report strong correlations between internalized weight stigma and body dissatisfaction (12, 20). Further, our results suggested that the strength of this relationship differs between sample population (clinical vs. community/college) as well as by BMI. Our results also suggest that measures of internalized weight stigma as well as measures of body dissatisfaction would benefit from further scrutiny in future research to improve the measurement of research questions, interpretation of results, and application of findings.

In their interpretation of results, Romano et al. (20) state that the association between internalized weight stigma with related concepts lowers the confidence in the literature pertaining to internalized weight stigma. However, our results suggest that consideration ought to be given to the measures of *both* internalized weight stigma *as well as* body dissatisfaction. According to the sociocultural model of disordered eating, a key predictor of body dissatisfaction is thin-ideal internalization (85), with thin-ideal internalization conceptualized as pressure from media, family, and peers to achieve a thin figure (86). Although researchers have recognized changes in the nature of the thin-ideal over time, such as the increasing emphasis of leanness *as well as* muscularity for both women and men (87), sociocultural beauty ideals that emphasize thinness are inherently anti-fat. Thus, anti-fat messaging is central to existing concepts of internalized weight stigma *as well as* body dissatisfaction, as evidenced by items on commonly used scales measuring both constructs. We argue that, as a construct, internalized weight stigma may be conceptualized as related to but distinct from thin-ideal internalization, due to its narrower focus on the internalization of weight-based stereotypes and negative beliefs, as opposed to the broader characteristics of ideal appearance in Western culture.

In considering the association between internalized weight stigma and body dissatisfaction it is important to acknowledge that, although the internalized weight stigma literature is relatively young compared to the literature on body dissatisfaction, this does not necessarily indicate a fatal flaw in this, or either, concept. Rather, a consideration of the histories and definitions of each construct over time may help to contribute to further discussion on whether the association between internalized weight stigma and body dissatisfaction is best described by concept creep or construct proliferation. In considering definitions and conceptualizations of internalized weight stigma, Durso and Latner (6) described it as distinct from body image, as it does not describe an individual's feelings about characteristics of the body beyond weight and shape. They also note that internalized weight stigma is distinct from self-esteem, but that it may contribute to an individual's self-esteem. Further, in describing the history of research on internalized weight stigma prior to the development of the WBIS, Durso and Latner (6) describe researchers as using measures of explicit weight stigma among higher-weight participants as indicators of internalized weight stigma, but that a distinction exists between weight stigma that is directed toward others vs. the self.

In a review of the history of body image literature, Grogan (3) highlights that research and clinical work related to body image and eating disorders until the 1980's reinforced ideas that the construct of body image only encompassed weight and shape. Grogan (3) also notes that, since the 1980's increased efforts have been made to include individuals across the lifespan as well as men in body image research. Despite increased efforts at inclusion in the literature, we assert that the connection between body image and weight remains strong, as evidenced by Grogan's (3) definition of body dissatisfaction as “negative evaluations of body size, shape, muscularity/muscle tone, and weight, and it usually involves a perceived discrepancy between a person's evaluation of his or her body and his or her ideal body” (p. 4).

Thus, while current definitions of body image and internalized weight stigma may differ, histories, definitions, and measures that focus on body dissatisfaction may not be distinct enough to provide a clear boundary between this construct and internalized weight stigma. Romano et al. (20) acknowledged support for the conceptualization by Meadows and Higgs (12) of internalized weight stigma and body dissatisfaction as tapping into a higher-order construct of body-related self-judgment, along with self-esteem. Given the anti-fat nature of both internalized weight stigma as well as body dissatisfaction, we believe that further research examining these constructs as part of a higher-order concept may be warranted.

## Strengths, Limitations, and Directions for Future Research

This study has several notable strengths. First, the addition of BMI and sample population as significant influences on the strength of the relationship between internalized weight stigma and body dissatisfaction provide important directions for future research. Second, the exploratory and confirmatory factor analyses investigating the conceptual overlap of two commonly used measures supports our assertion that conceptual overlap is present in the ways in which *both* internalized weight stigma and body dissatisfaction are measured. Finally, our sample in the empirical study consisted of female college students diverse in both age as well as race and ethnicity, suggesting our findings can be extended to a diverse population of women.

We also recognize three important limitations of this work. First, our exploratory and confirmatory factor analyses were limited to one common measure of each construct and does not fully explore the measurement overlap in all measures commonly used in the literature. Future research on the measurement overlap of more than two measures within the same sample will provide additional data to characterize the extent and nature of conceptual overlap in body image and internalized weight bias literatures. Second, our examination of measurement overlap was limited to a college sample and did not include a clinical sample of individuals engage in clinical weight management or treatment for eating disorders. Future research examining the measurement overlap of commonly used measures may benefit from the inclusion of both a clinical and community/college sample, given the results of our meta-analysis in study one. Third, we echo the limitations of the overall literature noted by Romano et al. (20) regarding key demographic features of participants in research published to-date. Further research is needed to better understand the conceptual overlap of internalized weight stigma and body dissatisfaction among individuals of different age ranges, gender identities, racial identities, and sexual orientations.

## Conclusion

Although both body dissatisfaction and internalized weight stigma are strongly related to the development and maintenance of disordered eating and clinical EDs, we call for the development of new, or refinement of existing, measures that lessen the degree of conceptual overlap between these constructs, thus improving the boundaries between these two concepts and ensuring measurement error is better accounted for (16). Such an improvement would allow researchers and clinicians to better tease apart the interactive and additive roles of body dissatisfaction and internalized weight stigma on disordered eating and other psychological outcomes, and to design strong intervention and prevention programs specifically tailored to each of these risk factors.

## Data Availability Statement

The raw data supporting the conclusions of this article will be made available by the authors, without undue reservation.

## Ethics Statement

The studies involving human participants were reviewed and approved by Florida International University Social and Behavioral Sciences. The patients/participants provided their written informed consent to participate in this study.

## Author Contributions

JS and SN conducted the systematic review and meta-analyses. JS collected and analyzed the data from study 2. SR-M assisted in writing earlier versions of the manuscript. All authors contributed to the study development and approved the submitted version.

## Funding

This work was funded by Social Sciences and Humanities Research Council (SSHRC) Grant 435-2018-0116.

## Conflict of Interest

The authors declare that the research was conducted in the absence of any commercial or financial relationships that could be construed as a potential conflict of interest.

## Publisher's Note

All claims expressed in this article are solely those of the authors and do not necessarily represent those of their affiliated organizations, or those of the publisher, the editors and the reviewers. Any product that may be evaluated in this article, or claim that may be made by its manufacturer, is not guaranteed or endorsed by the publisher.
