# Corrigendum: Examining the Conceptual and Measurement Overlap of Body Dissatisfaction and Internalized Weight Stigma in Predominantly Female Samples: A Meta-Analysis and Measurement Refinement Study

**DOI:** 10.3389/fgwh.2022.928488

**Published:** 2022-05-16

**Authors:** Jessica F. Saunders, Sarah Nutter, Shelly Russell-Mayhew

**Affiliations:** ^1^Hiatt School of Psychology, Clark University, Worcester, MA, United States; ^2^Department of Educational Psychology and Leadership Studies, University of Victoria, Victoria, BC, Canada; ^3^Werklund School of Education, University of Calgary, Calgary, AB, Canada

**Keywords:** body dissatisfaction, internalized weight stigma, internalized weight bias, measurement, concept proliferation

In the original article, there was an error. During the proofing stage, the UK spelling we used in our systematic review search was changed to US spelling

A correction has been made to **Method, Search Strategy and Eligibility Criteria**, Paragraph one:

“In conducting our search, we examined the search strategies of two previous systematic reviews on internalized weight stigma (1, 20), and utilized the search terms for internalized weight stigma that were first identified by Pearl and Puhl (1) and also used by Romano et al. [(20): weight bias internalization; weight bias internalisation; internalized weight bias; internalised weight bias; internalized weight stigma; internalised weight stigma; self-directed weight stigma; self-directed weight bias; weight self-stigma]. The search was conducted in seven databases on January 31, 2022, by the second author: PubMed, SCOPUS, Science Direct, EMBASE, PsychINFO, CINAHL, and Web of Science. Studies were included if they were written in English, tested human participants, quantitatively measured both body dissatisfaction (defined as measuring dissatisfaction with appearance, weight, and/or shape) and internalized weight stigma, and were peer reviewed. The search yielded 585 abstracts after the removal of duplicates (*n* = 1,032), which were screened by the first and second authors. Of the 76 papers that were identified for full-text screening, 48 were included in the systematic review and 43 in the meta-analysis. Given the small number of studies examining these constructs in child and adolescent samples (*n* = 5), we included these in the systematic review for descriptive purposes but not the meta-analysis. Papers were excluded from the systematic review and meta-analyses (*n* = 28) for the following reasons: (1) not measuring both constructs in question (*n* = 4), (2) conference abstracts, theses, and dissertations (*n* = 8), (3) or the correlation not being available from the study authors (*n* = 16). The search structure and process are summarized in Figure 1. Notably, we identified an additional 20 papers that were not included in the meta-analysis conducted by Romano et al. (20), two of which were published online after October 2021, when their search was conducted.”

The authors apologize for this error and state that this does not change the scientific conclusions of the article in any way. The original article has been updated.

## Publisher's Note

All claims expressed in this article are solely those of the authors and do not necessarily represent those of their affiliated organizations, or those of the publisher, the editors and the reviewers. Any product that may be evaluated in this article, or claim that may be made by its manufacturer, is not guaranteed or endorsed by the publisher.

